# X-ray Tomography Unveils the Construction Technique of Un-Montu’s Egyptian Coffin (Early 26th Dynasty)

**DOI:** 10.3390/jimaging8020039

**Published:** 2022-02-07

**Authors:** Fauzia Albertin, Maria Pia Morigi, Matteo Bettuzzi, Rosa Brancaccio, Nicola Macchioni, Roberto Saccuman, Gianluca Quarta, Lucio Calcagnile, Daniela Picchi

**Affiliations:** 1 Department of Physics and Astronomy “Augusto Righi”, University of Bologna, 40127 Bologna, Italy; matteo.bettuzzi@unibo.it (M.B.); rosa.brancaccio@unibo.it (R.B.); 2 INFN National Institute for Nuclear Physics, Division of Bologna, 40127 Bologna, Italy; 3 CNR—IBE Institute of BioEconomy, National Research Council, Sesto Fiorentino, 50019 Florence, Italy; nicola.macchioni@ibe.cnr.it; 4 Independent Scholar, Marsciano, 06055 Perugia, Italy; rosaccuman@gmail.com; 5 CEDAD—Centre of Applied Physics, Dating and Diagnostics, Department of Mathematics and Physics “Ennio de Giorgi”, University of Salento, 73100 Lecce, Italy; gianluca.quarta@unisalento.it (G.Q.); lucio.calcagnile@unisalento.it (L.C.); 6 Archaeological Museum of Bologna, Egyptian Collection, 40124 Bologna, Italy

**Keywords:** egyptian coffin, X-ray tomography, in situ analysis, non-invasive investigations, radiocarbon dating, wood identification

## Abstract

The Bologna Archaeological Museum, in cooperation with prestigious Italian universities, institutions, and independent scholars, recently began a vast investigation programme on a group of Egyptian coffins of Theban provenance dating to the first millennium BC, primarily the 25th–26th Dynasty (*c.* 746–525 BC). Herein, we present the results of the multidisciplinary investigation carried out on one of these coffins before its restoration intervention: the anthropoid wooden coffin of Un-Montu (Inv. MCABo EG1960). The integration of radiocarbon dating, wood species identification, and CT imaging enabled a deep understanding of the coffin’s wooden structure. In particular, we discuss the results of the tomographic investigation performed in situ. The use of a transportable X-ray facility largely reduced the risks associated with the transfer of the large object (1.80 cm tall) out of the museum without compromising image quality. Thanks to the 3D tomographic imaging, the coffin revealed the secrets of its construction technique, from the rational use of wood to the employment of canvas (*incamottatura*), from the use of dowels to the assembly procedure.

## 1. Introduction

Scientific analyses of works of art are widely used in the diagnostic phase prior to the restoration intervention, giving fundamental information about the materials and construction techniques and providing guidance for the best possible conservation methodology. In addition to this, such analyses help to eventually disclose the fingerprints and the approaches of the artist or craftsman responsible for their creation. In recent years, impressive developments in techniques for analyses of cultural heritage assets (with regard to both the range of techniques applied and the instruments used) have made many methods available on site and enabled a non-invasive approach.

This is the case in multispectral imaging techniques that give a broad picture of the artefact [1], revealing underdrawings [2]; restored surfaces; and, in some cases, the nature of the pigments [3,4]. The combination of these techniques with other investigations, such as XRF [5,6] and SEM-EDS for the elemental analysis of pigments [7], and FT-IR and Raman spectroscopy for the study of organic components [8,9], make it possible to identify constituent materials without the need for sampling [10,11]. Moreover, emerging techniques such as THz imaging [12,13,14] allow for non-invasive investigation of the external stratigraphy of the objects.

For some extremely sensitive, peculiar, or in-depth analyses, the use of large facilities like synchrotrons [15,16] or neutron spallation sources [17,18,19] is still required. Nevertheless, nowadays, most investigations are available in situ, inside museums or restoration labs, avoiding any risky transport of valuable and/or bulky artefacts and works of art.

Among the latter techniques, it is worth mentioning X-ray computed tomography (X-ray CT) [20,21], which allows for non-invasive investigation of the entire structure of objects, thus enabling the identification of the different constituent materials and the study of the construction technique [22,23]. X-ray CT is a versatile technique that can be applied to a wide range of cultural heritage artefacts of different sizes and compositions [24].

In this paper, we present the results of an investigation carried out on a particularly interesting case study: the anthropoid wooden coffin of Un-Montu, belonging to the Egyptian collection of the Bologna Archaeological Museum (Inv. MCABo EG1960). The integration of radiocarbon dating, wood species identification, and CT imaging enabled the construction technique to be unveiled, from the use of canvas for the so-called *incamottatura* below the painting layers to the selection and assembly of the wooden planks used to create the anthropomorphic shape of the coffin. 

The coffin was restored by the Bologna Archaeological Museum in 2018, as part of the Intesa Sanpaolo *Restituzioni* project [25]. The conservation project was preceded by archival research and a multidisciplinary study of the coffin aimed at reconstructing the stages of its deterioration. That deterioration derived from both the collecting vicissitudes that brought it from Egypt to Bologna and from techniques and materials adopted in the past for conservation purposes. In particular, the conservation and aesthetic interventions visible on the artefact were partly attributable to a restoration performed in the 1960s and partly attributable to previous undocumented treatments.

The information obtained was fundamental to determine the type of interventions with which an object transferred to the museum’s warehouses in the 1990s was returned to the public in 2018. This project also represented the ideal opportunity to codify the most appropriate methodological approaches to the study of the wooden structure and assembly techniques of another four anthropoid coffins from the Theban area, dating to the 25th–26th Dynasty (*c.* 746–525 BC). These represent one of the most prestigious classes of materials in the Bologna collection.

### 1.1. The Anthropoid Coffin of Un-Montu, from Egypt to Bologna

The anthropoid wooden coffin of Un-Montu, an Egyptian dignitary whose name reveals devotion to the Theban god Montu, arrived in Bologna in 1861 via testamentary bequest of the Bolognese painter Pelagio Palagi (1775–1860), who died in Turin in 1860. Palagi collected 3109 Egyptian antiquities over a period of twenty years, from about 1825 to 1845, thus becoming the only private individual of the time able to compete with statesmen, governments, and sovereigns involved in the creation of the great European museums. From whom and when Palagi acquired this artefact is unknown. As far as we were able to discern, the Un-Montu coffin was mentioned for the first time in the inventory of the Palagi museum in Milan [26,27], which was drawn up in 1860 after the painter’s death before his Egyptian collection was moved to Bologna. The inventory describes it as a painted and varnished coffin, which is useful information for reconstructing its conservation history. 

**Figure 1:**
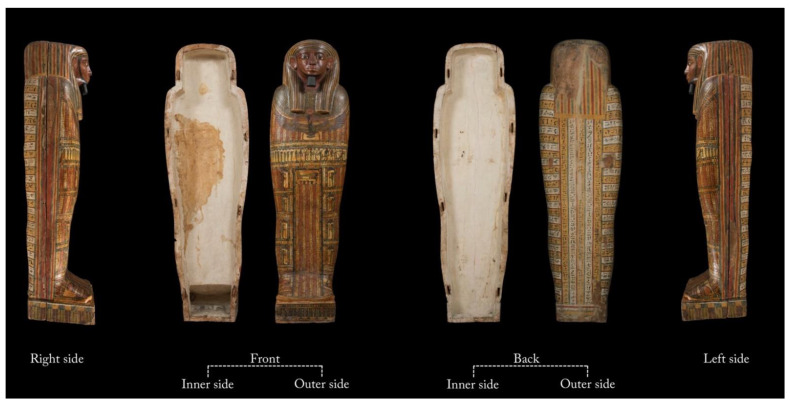
Inner and outer sides of Un-Montu’s coffin (Inv. MCABo EG1960).

The Un-Montu coffin is 183 cm long, 54 cm wide, and 53 cm deep (Figure 1). The anthropoid figure with its dorsal pillar and pedestal gives the coffin a sculptural shape, with the appearance of a mummy wrapped in a linen shroud and raised to a vertical position. This bivalve anthropoid form characterizes the Theban inner coffins of the 25th–26th Dynasty (*c.* 746–525 BC).

The head is covered by a tripartite wig: a very large rear band and two longer, narrower, and rounded lappets at the front sides with alternating yellow and blue parallel stripes. The face and its protruding ears are coloured in brown, the lips are coloured in red-brown, and the short beard on the chin is coloured in dark blue. 

On the basis of stylistic comparisons, the frontal body-field of this inner coffin corresponds to ‘Design 3’ of John Taylor’s classification of the 25th–26th Dynasty inner Theban coffins [28]. An enveloping and polychrome *usekh* collar, consisting of geometric and floral elements, covers the upper part of the chest; immediately below, the goddess Nut sits on a *nbw* sign. 

On the upper abdomen, the lid is painted with a horizontal register, representing the weighing of the heart and the presentation of the deceased to a series of deities and, on the legs, with a central panel consisting of seven columns of inscriptions running to the feet, which is flanked by a symmetrical sequence of deities inside shrines and texts on a white background. On the top of the panel, there is a mummy on a bier. On the feet, the central panel is flanked by two *udjat* eyes. 

**Figure 2:**
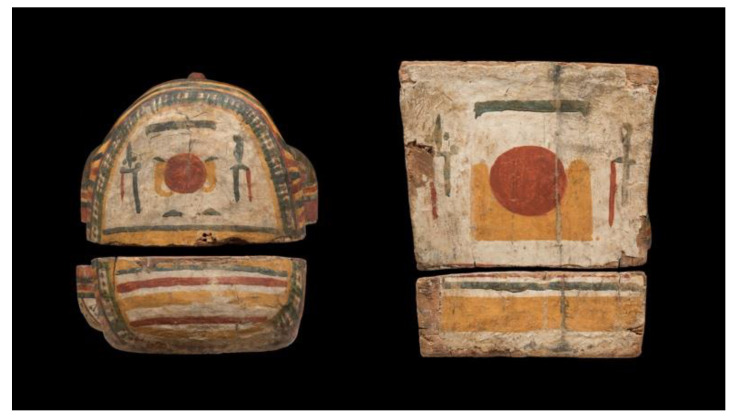
Head end/wall and foot board of Un-Montu’s coffin.

At the foot board and at the head end of the lid, two different moments of the solar cycle are represented, in order to associate the deceased with the eternal daily rebirth of the celestial star (Figure 2). 

A dark-amber varnish covers a great part of the outer side of the lid. The outer side of the box features vertical columns of inscriptions on the pillar and horizontal lines of texts on its sides, which are painted in black on white and yellow backgrounds; this decorative layout corresponds to ‘Design 2’ of Taylor’s classification of the 25th–26th Dynasty inner Theban coffins [28]. The inner sides of the lid and box are painted in white. Because the wings of Nut are subdivided into four sections and there is a repeating frieze of *ankh-neb-was* symbols on the front of the pedestal, the coffin is presumed to date to the early 26th Dynasty (664–525 BC).

### 1.2. Coffin X-ray Tomography

X-ray computed tomography is a non-invasive and non-destructive 3D imaging technique capable of revealing crucial information about the manufacturing and assembly techniques of complex objects, such as the Un-Montu coffin. The CT technique investigates the entire volume of an artefact, providing scholars and restorers with knowledge of inner hidden details such as joints and dowels [20,21,29], the presence of different materials [10,30], the construction technique [22,31,32], or the casting process [33,34], and helps to elucidate possible restoration treatments and preservation conditions [10,35,36].

Numerous scientific papers have focused on the CT analysis of Egyptian mummies by means of medical scanners [37,38,39,40,41,42], but CT has been applied to coffins or other wooden artefacts realized by ancient Egyptian craftsmen in a few studies. 

For example, a new generation multi-slice CT was used by Amenta [43] and Longo et al. [44] to investigate an Egyptian wooden mummy board from the Vatican collection (Inv. MV25022). Their research was carried out in the framework of *The Vatican Coffin Project* [45], a large international and interdisciplinary study launched by the Vatican Museums, which is focused on Third Intermediate Period Egyptian coffins. The use of two distinct techniques of image post-processing, multi-planar reconstruction (MPR) and the volume rendering technique (VRT), allowed researchers to evaluate the state of conservation of the mummy board, to determine how the wooden object was manufactured, to reveal evidence of a nineteenth-century intervention of consolidation, and to hypothesize that the wooden planks were reused. 

Re et al. [46] highlighted the importance of tomography in the study of wooden artefacts, making a comparison with radiography in the case of Taiefmutmut’s coffin lid. Even though the artefact was quite simple in geometry and constituent materials, the results obtained with the two techniques were noticeably different. In particular, tomography provided more information, not only on the state of conservation, but also on manufacturing techniques and previous restorations. However, it should be noted that the tomography of an entire coffin generally requires much longer acquisition times (potentially many hours) than radiography [1]. 

Among the scientific projects concerning the study of ancient Egyptian artefacts, it is also worth mentioning *The Egyptian Coffin Project* of the Fitzwilliam Museum in Cambridge [47]. In 2014, the Museum began a broad, interdisciplinary research effort into its collection of Egyptian coffins and coffin fragments, involving the application of advanced analytical and imaging techniques, including radiography and computed tomography. Their research provided remarkable insights into the coffins’ construction and decorative programme, and all the results are freely available to the general public [47]. Most often, in the past, only X-ray radiography was employed as part of a multi-technique approach [48,49,50,51,52].

Despite the invaluable capabilities of the tomographic technique, a wider use of CT in the field of cultural heritage is limited by two main obstacles: first, most of the existing CT scanners are optimized for the human body, and second, the key requirement for the safety of cultural heritage objects, i.e., the in situ availability of the analyses, is difficult—if not impossible—to meet. Alternate approaches, such as flexible CT facilities [53], equipment for industrial CT [54], and custom facilities [55] installed in specialized labs inside museums or restoration centres [56], may be feasible. These solutions eliminate the risks associated with artefact transportation and make X-ray tomography available as a routine analysis. However, only major institutions have these advanced labs. 

To overcome these limitations, the *X-ray Tomography Group* of the Department of Physics and Astronomy of Bologna University designed and developed several transportable CT systems devoted to cultural heritage analysis [22,57,58,59]. These systems are characterized by high flexibility and versatility and allow the tomographic investigation of a wide range of objects with varied shapes and sizes, as well as an optimized acquisition on a case-by-case basis.

## 2. Materials and Methods

### 2.1. Wood Species Identification

The collecting of samples from Un-Montu’s coffin followed the guidelines described in the Italian technical standard UNI 11118:2004 (Cultural heritage–Wooden artefacts–Criteria for the identification of wood species).

After the preliminary macroscopic observations, 18 samples were drawn from different portions of the wooden structure of the coffin, from both the box and lid. The samples were boiled in water in order to facilitate cutting them into thin sections according to the three main anatomical directions of wood (cross, longitudinal radial, and longitudinal tangential). The sections, obtained with manual cutting, were mounted with a few drops of glycerol on microscope slides and covered with coverslips. Observations were made with an optical microscope (Leica DM LB2). Identifications were obtained through observation of the anatomical features.

### 2.2. ^14^C Dating

A wood sample, obtained from a mortise in the left side of the coffin box, was submitted to be radiocarbon dated at the Centre of Applied Physics, Dating, and Diagnostics (CEDAD) of the University of Salento, Lecce, Italy. The sample (Laboratory code LTL17852A) was preliminarily analysed at the optical microscope to highlight possible sources of contamination and then processed in order to prepare it for AMS (accelerator mass spectrometry) analysis.

The sample underwent the standard acid–alkali–acid protocol used at CEDAD for wood samples, aimed at removing possible contaminations. It was then dried at 60 °C overnight. The purified, dried material was then vacuum-sealed in quartz tubes together with copper oxide and silver wool and combusted with CO_2_ at 900 °C for 4 h [60]. The obtained carbon dioxide was cryogenically purified and reduced at 600 °C to graphite using hydrogen as a reducing agent and iron powder as a catalyst. The obtained ~2 mg graphite/iron mixture was then pressed in aluminium target holders to be used as cathodes in the sputtering ion source of the AMS system, based on a 3 MV Tandetron Type accelerator (Mod. HVEE 4130HC) [61]. The ^14^C/^12^C isotopic ratio was then measured with the system, from which, after corrections for isotopic fractionation and background, the conventional radiocarbon age was calculated [62]. The conventional radiocarbon age was then calibrated to calendar age using the last internationally accepted INTCAL20 calibration curve, valid for atmospheric data, and the software OxCal Ver 4.4 [63].

### 2.3. CT Scanning

The tomography system used for Un-Montu’s coffin investigation at the Bologna Archaeological Museum was designed for medium/high-resolution 3D imaging (minimum voxel size 100 μm) of objects up to 150 cm in size. Moreover, the fast acquisition process—each tomographic frame requires only 3 min—made the system optimal for the CT analysis of large objects. 

The system has an open design; the main components (source, rotation stage, and detector) are unbonded and unshielded. X-ray projections are acquired in cone-beam geometry by rotating the object over 360° between the source and the detector. Radiation safety is guaranteed by a no-go zone defined on the basis of the criteria established by a qualified expert in radiation protection. The investigation was conducted overnight.

**Figure 3:**
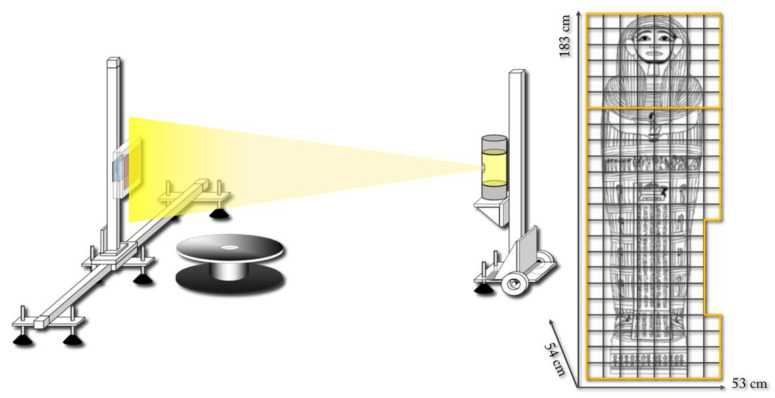
Tomographic acquisition set-up: on the left, the layout of the CT facility; on the right, the tile-scanning grid used to collect the entire tomographic data sets.

The facility, the layout of which is shown in Figure 3 (left), was equipped with an X-ray tube (Smart EVO 200D, YXLON International GmbH, Hamburg, Germany, 30–200 kV, and maximum current of 6 mA), a fast flat panel detector (C10900D, Hamamatsu Photonics K.K., Hamamatsu, Japan, with a CsI/TI scintillator, 1216 × 1232 pixel, and 100 μm pixel size), and a heavy load rotational stage (mod. PRS200, Physik Instrumente (PI) GmbH & Co., Karlsruhe, Germany). 

To acquire the tomographic data of the entire object, the so-called tile-scanning technique was used: two orthogonal translation axes moved the detector on an X–Y plane, while a Z-translation axis moved the source. According to this scanning modality, the acquisition of the tomographic data was performed step-by-step by moving the detector on a virtual grid to cover the entire radiographic projection of the object with a certain number of different frames (Figure 3 right). For each detector position in the grid, a series of 900 projections was acquired. Considering that the height of Un-Montu’s coffin overcame the range covered by the Z-axis, the acquisition was divided into two sessions. The first covered from the foot board to the upper chest and the second from the upper chest to the head wall. 

To achieve the full coffin’s CT imaging, a total of 184 tomographic data sets were acquired. The main parameters for the CT scanning are summarized in Table 1. All the acquisition process required several hours—3 min for each set of projections—for a total of 115 GB of data.

The tomographic reconstruction was performed with a developed in-house software [64,65] based on the cone-beam algorithm of Feldkamp, Davis, and Kress (FDK) [66]. 

Owing to the tile-scanning procedure, the tomographic reconstruction of the entire volume was carried out by successive steps. For each horizontal line of the grid, the projections acquired at each detector position were processed with dark image subtraction and flat field correction and then stitched. The horizontal sections of the coffin volume were then reconstructed, taking care of the eventual presence of ring artefacts and their removal. Lastly, the so-reconstructed partial coronal slices of the coffin were vertically stitched to create the final volume. 

The 3D rendering was performed by means of VGStudio Max (Volume Graphics GmbH) and the open-source software 3D Slicer [67]. The CT images were elaborated and displayed using the open-source software Fiji [68] and Gimp [69]. 

**Table 1:** Main acquisition parameters for the CT analysis of Un-Montu’s coffin.

Acquisition Parameters	
X-ray tube voltage	150 kV
X-ray tube current	2.2 mA
X-ray beam filter	Al (2 mm)
Source-detector-distance (SDD)	345 cm
Source-object-distance (SOD)	290 cm
Object-detector-distance (ODD)	55 cm
Magnification	1.19
Detector frame rate	5 fs
Detector pixel size	100 μm
Binning	2 × 2
Number of projections	900
Reconstructed voxel size	168 μm

## 3. Results

### 3.1. Coffin Woods

Thanks to the focused sampling of the wooden structure, two timbers were identified. All the samples taken from the coffin planks were found to be of *Ficus sycomorus*. All the connecting elements, both dowels and tenons, were found to be of *Tamarix* sp (probably *T. aphylla*). One sample from a tenon was too small to obtain any useful information. Sampling areas and identification of the 18 wooden samples are detailed in the Supplementary Materials—Wood Species (Figures S1 and S2).

Both woods are indigenous; the local fig tree (*Ficus sycomorus*) has been frequently found in the identification of wood used for Egyptian coffins [70,71,72,73,74], particularly for wide and long coffin planks, as in the present analysis. Fig tree wood is light and easy to work with, an important feature for the tools available in that period. Tamarisk wood (*Tamarix* sp.) has been frequently found in dowels and other joining systems like tenons [73,74]. Most Egyptian tamarisks are small trees or shrubs, except *T. aphylla*. It is likely that the tamarisk wood identified in many samples taken from coffins could come from the latter species.

### 3.2. Coffin Dating

**Figure 4:**
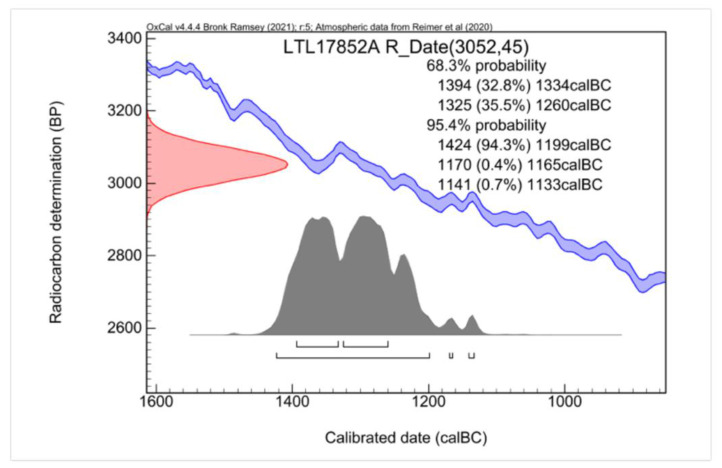
The results of the ^14^C dating: in blue, the calibration curves (plus and minus one standard deviation); in red, the radiocarbon date; and in grey, the calibrated date.

As mentioned above, for the ^14^C dating, a wooden sample was taken from a mortise in the left side of the coffin box. A radiocarbon age of 3042 ± 45 BP was measured for the sample, which was then calibrated to the range 1424–1199 BC with a probability of 94.3% (Figure 4).

This analysis did not confirm the chronological attribution on a stylistic basis of Un-Montu’s coffin to the early 26th Dynasty, but did confirm the well-attested practice of reusing wood to make coffins, very common from the 19th to 22nd Dynasty (1292–730 BC) [75,76,77]. The results listed in Figure 4 showed that the wooden specimen dated to the New Kingdom, 18th–19th Dynasty (1539–1186 BC). In this case, unfortunately, it was impossible to establish whether only the left side plank of the box was reused, or whether other planks were, as well. 

### 3.3. The Wooden Structure

The rendering of the entire coffin tomographic data enabled comprehension of its constituent elements and the construction techniques used in its manufacture. 

**Figure 5:**
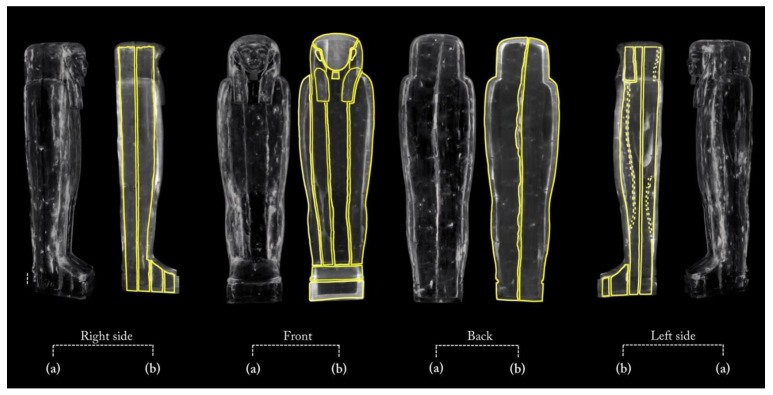
(**a**) 3D rendering of the sides of Un-Montu’s coffin and (**b**) visualization of its planks on each side.

Figure 5 shows the main views from the outside of the object (front, back, and left and right sides), using two different display modes. 

The 3D rendering of the coffin, in semi-transparent grey levels (Figure 5a), highlighted the complex wooden structure of the coffin, made of planks and a plaster-like material (stucco). The latter was clearly used to fill, model, and smooth the planks’ connections, as well as to cover the dowel holes and the narrow cracks in the wood. 

Then, to better investigate this complex wooden structure, the tomographic slices related to each side were summed and the different wooden planks were outlined with a yellow line, as shown in Figure 5b.

- The floor board

**Figure 6:**
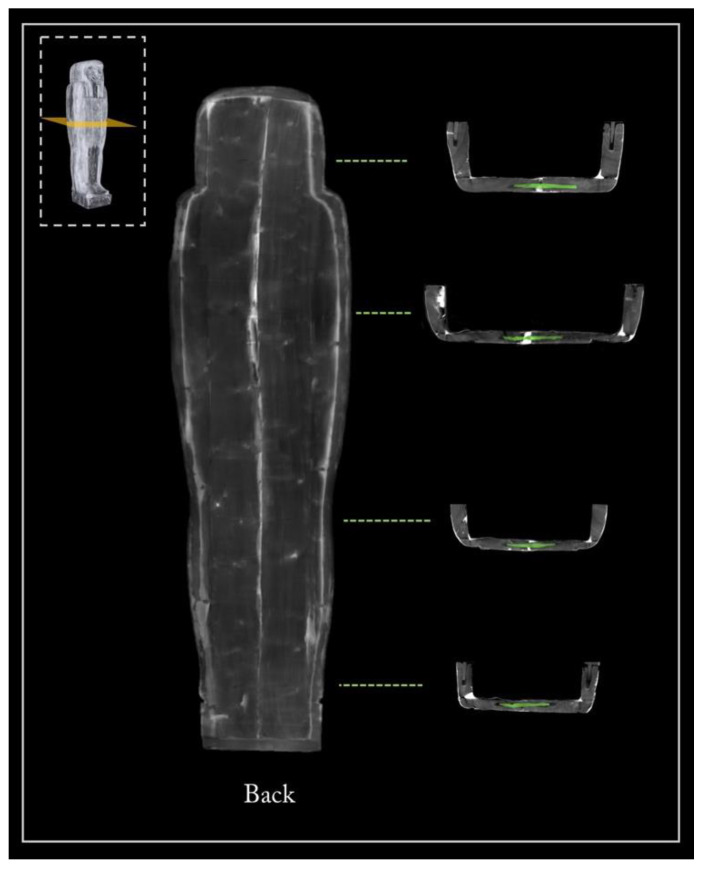
3D rendering of the underside of the floor board: in green, the four dowels joining the two planks.

As for the coffin box, the floor board consists of two large planks. Each plank has the pith off-centred towards the outside, indicating that the planks were wider and presumably reduced by processing after assembly. The ‘recto’ side (the face of the plank closer to the pith) of the plank is correctly facing faced outward. The two planks are juxtaposed and connected by four round dowels (Figure 6).

The contact edges were worked and shaped by abrasion or axe to be able to adhere, with the maximum saving of material and time. The right plank shows an area with uneven and fast-growing wood and shrinkage checks. The two planks apparently come from the same stem; the right plank seems to be obtained from the back of the left one. This is visible from the rings’ path, taking into account the working wastes. The natural left-handed curvature of the stem is exploited to obtain a better coupling of the two planks.

- The front of the lid

**Figure 7:**
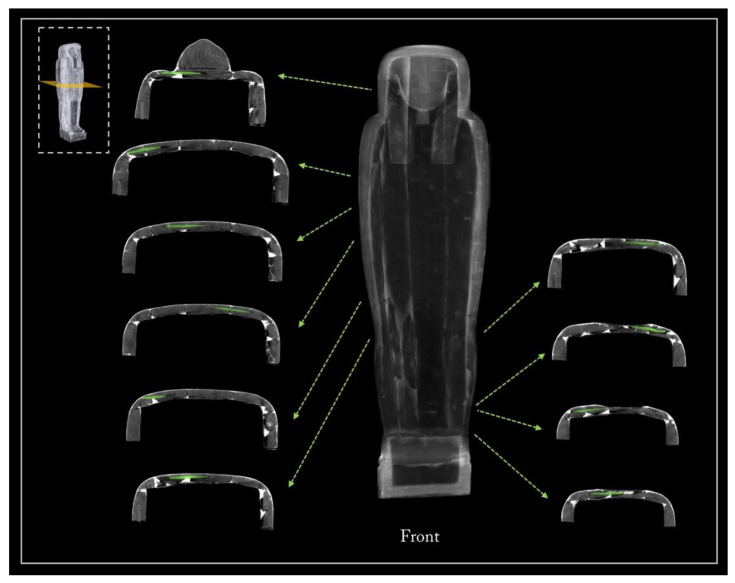
3D rendering of the front of the lid: in green, the numerous dowels joining the four planks.

**Figure 8:**
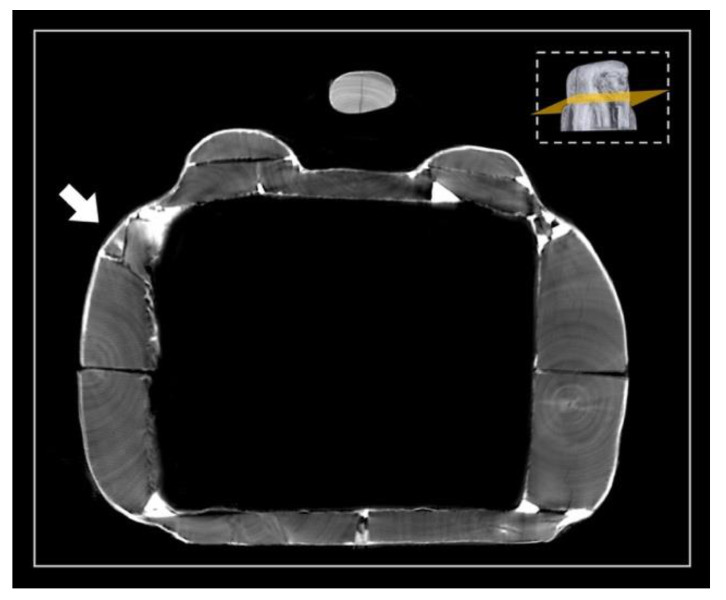
Axial slice showing the small insert with a triangular section.

The front of the lid consists of four narrower planks, obtained from small diameter stems and joined by round dowels (Figure 7). The central plank decreases in width, owing to natural tapering, and moves slightly to the left, proceeding downward. The plank on its left is integrated with a small insert, with a triangular section placed next to the upper chest (Figure 8), while the two planks on its right were probably of the same size in the assembling phase, and were reduced during the final modelling. These two planks are tangentially cut and are barely sufficient in some portions. The most central one seems to be positioned upside down, with the recto facing outwards, unlike the others, and in the lower part, it shows large areas of grouting and several inserts applied upside down. Several defects clearly visible on the plank would be compatible with a tangential cut from a stem characterized by several irregularities, typical of the identified tree (*Ficus sycomorus*).

- The coffin face

**Figure 9:**
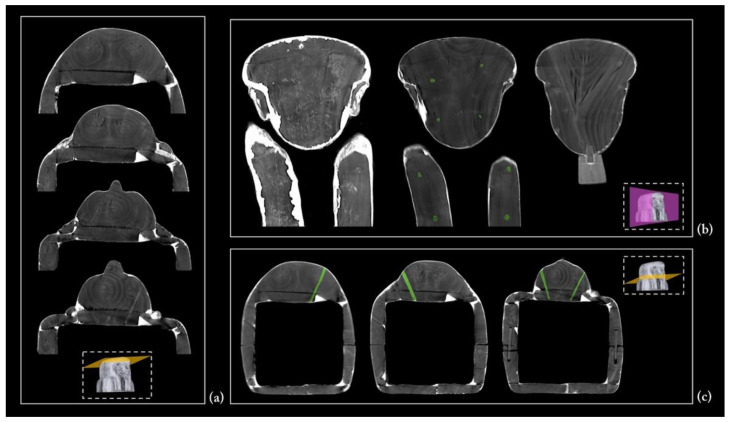
(**a**) The two splayed piths in the wooden block of the face; (**b**,**c**) face, ears, beard, and wig’s lappets joined by means of glue, plaster-like materials, and dowels.

The face, the ears, the beard, and the wig lappets are separate wooden elements, applied afterwards to the coffin lid structure. The presence of two splayed piths reveals that the face applied on the planking of the lid was obtained from a block of wood coming from the main branching area of the trunk (Figure 9a). The face and wig lappets are connected to the lower planks by round dowels, four and two for each lappet, respectively. Interestingly, the CT investigation also revealed the presence of glue between these latter and the lower planks, probably to enforce their stability (Figure 9b,c). The ears are fixed with a thick layer of the plaster-like material and the beard is joined to the chin by means of a square dowel (Figure 9b).

- The side planks

**Figure 10:**
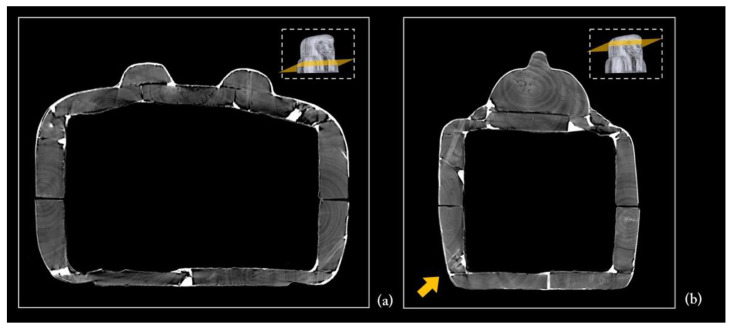
(**a**) Axial slice showing the matching growth rings of the side planks; (**b**) partial detachment of the growth rings, ring-shake (indicated by the orange arrow).

**Figure 11:**
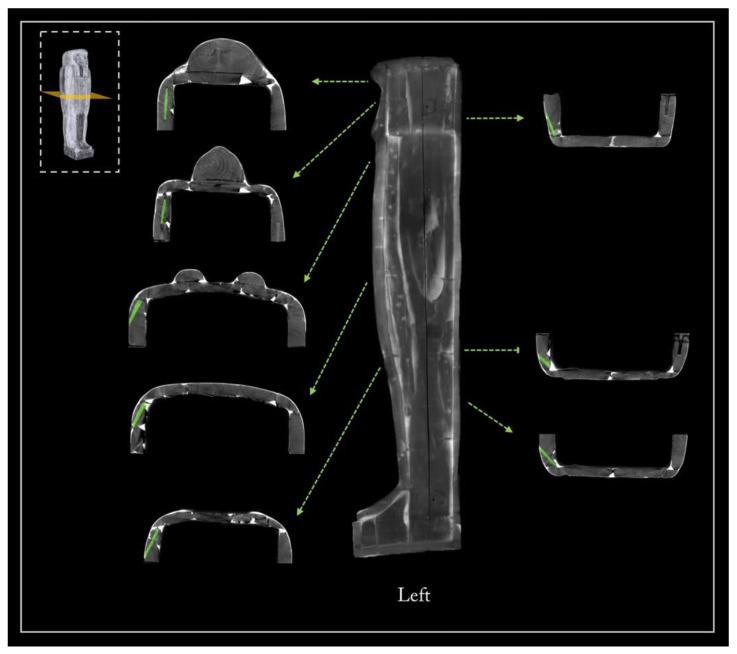
3D rendering of the left side of the coffin, lid, and box. In green, the numerous dowels joining the planks.

It is worth noting that each side of the coffin derives from a single large plank, as evident from the matching growth rings (Figure 10a). The side planks are rather thick and high quality compared with the other planks. This choice was likely owing to the needs both to realize, at a later time, the mortices for the coupling tenons and to work them with the available technologies for the final shaping. The need to use two thick planks was also justified by the fact that these formed the perimeter frame of the box and lid and allowed a better internal finishing work thanks to the robustness of the structure. The rendering also unveils growth defects of the trunk that are visible on the left side plank, of both the box and lid (Figure 5, left side), and are due to a partial detachment of the growth rings, i.e., a ring-shake (Figure 10b). This plank reveals a more evident decay than the right side one. Moreover, on the left side, at the height of the head, there is an added block to widen or compensate for the width of the plank (Figure 5, left side; Figure 10). All these elements are connected by round dowels (Figure 11).

- The upper head wall

**Figure 12:**
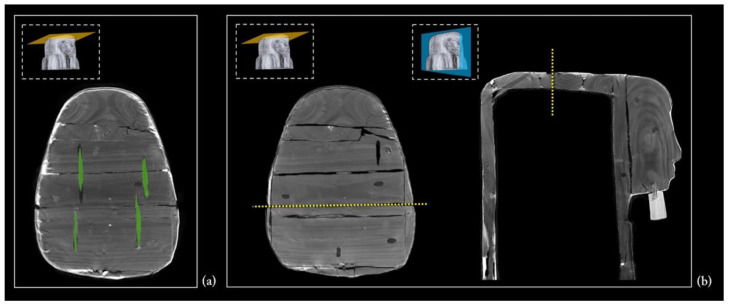
(**a**) The dowels connecting the three planks of the head wall; (**b**) axial and sagittal slices showing the cut of the central plank.

The upper part of the coffin head was assembled using smaller planks, always joined by means of round dowels. More precisely, it is made of three planks joined with two dowels for each joint (Figure 12a). The central plank was cut after the assembling of this planking, as demonstrated by the yellow dashed line in Figure 12b.

- Feet, pedestal, and foot board

**Figure 13:**
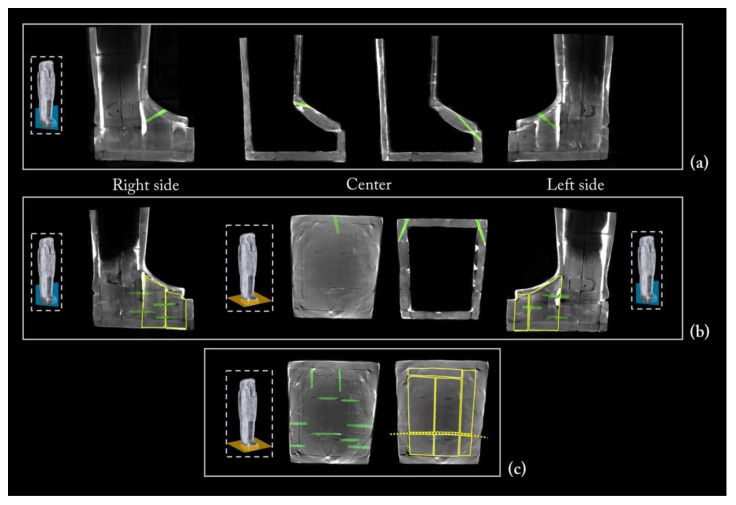
(**a**) Sagittal sections of the feet and pedestal showing the connecting dowels; (**b**) the wooden small planks that complete the front of the pedestal and the coffin sides with related dowels; (**c**) the planking of the foot board.

The feet and pedestal are made of smaller wooden elements that complete the front of the lid and both of the coffin sides. As shown in Figure 13a, on the front, the feet are made up of a rather thick siding board, while on each side, two smaller planks complete the pedestal (Figure 13b). The latter is closed on the front by another small plank. All the connections are made by round dowels. 

The foot board consists of four planks, two of the same width and two narrower. Each plank is joined with two dowels for each joint, according to a methodology adopted in other parts of the structure (Figure 13c). In this planking, the grain direction runs from the floor board to the lid. After the assembly, this planking was cut, as demonstrated by the yellow dashed line in Figure 13c.

- Tenons and mortises

**Figure 14:**
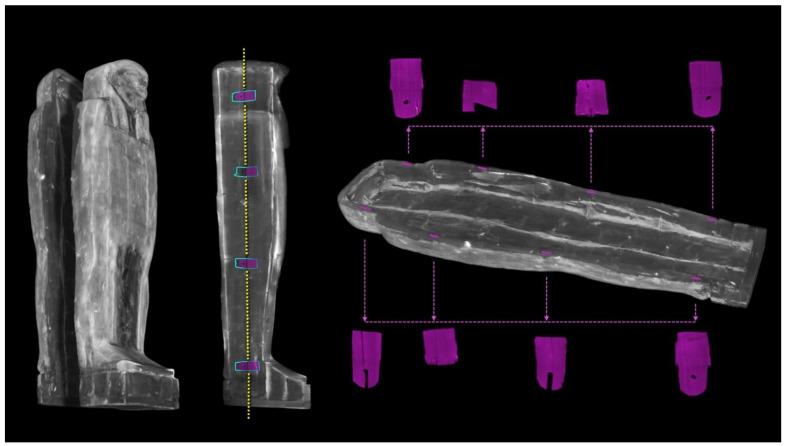
The 3D rendering of the opened coffin (left) and analysis of its closing system with the eight tenons virtually extracted (in purple).

The CT digitization of the coffin enabled a better analysis of the joining system of its halves (Figure 14). The box and lid flat edges are characterized by four mortises per side to hold a tenon. The ‘virtual extraction’ of the tenons from the mortises enables a better understanding of their shapes and the way they fit in the mortises. 

Only part of the eight tenons retains their original shape and is traversed by locking dowels inserted through corresponding holes in the box wall. The forced opening of the coffin, from which the mummy was extracted in the past, certainly caused the breaking of the tenons and their partial reshaping.

### 3.4. The Coffin’s Surface Layers

The CT analysis also added further information to the results of the multi-technological analytical protocol performed on the painted surface of Un-Montu’s coffin (the results of this study will be soon published in a dedicated paper).

A few microsamples, taken for a deeper insight into the composition and sequence of ground and paint layers by means of optical microscopy, SEM-EDX, and FTIR spectrometry, revealed the overlapping of three different surface layers. The first one corresponds to the canvas ground layer, while the second corresponds to the linen canvas itself (*incamottatura*), and the third to the ground layer of the polychrome layout. 

**Figure 15:**
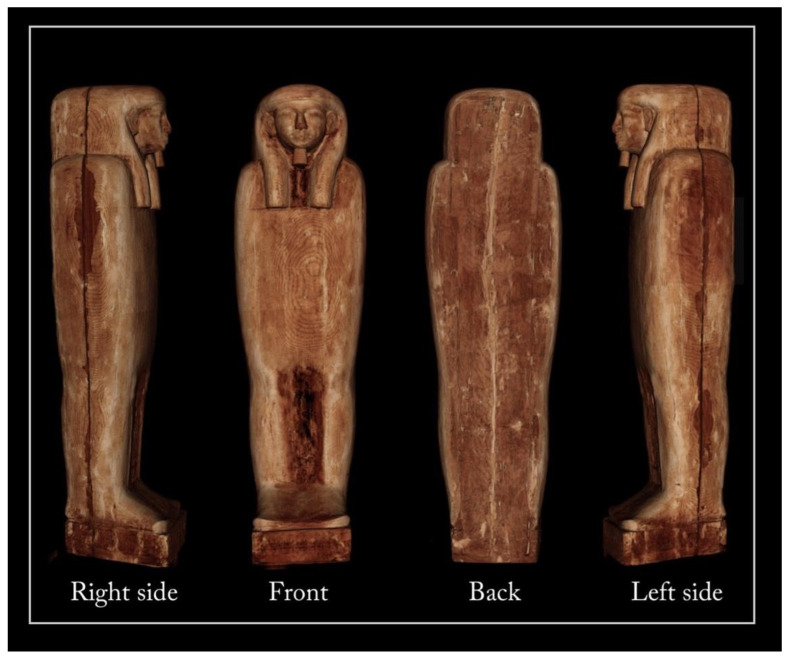
The stucco on the outer surface of the coffin.

**Figure 16:**
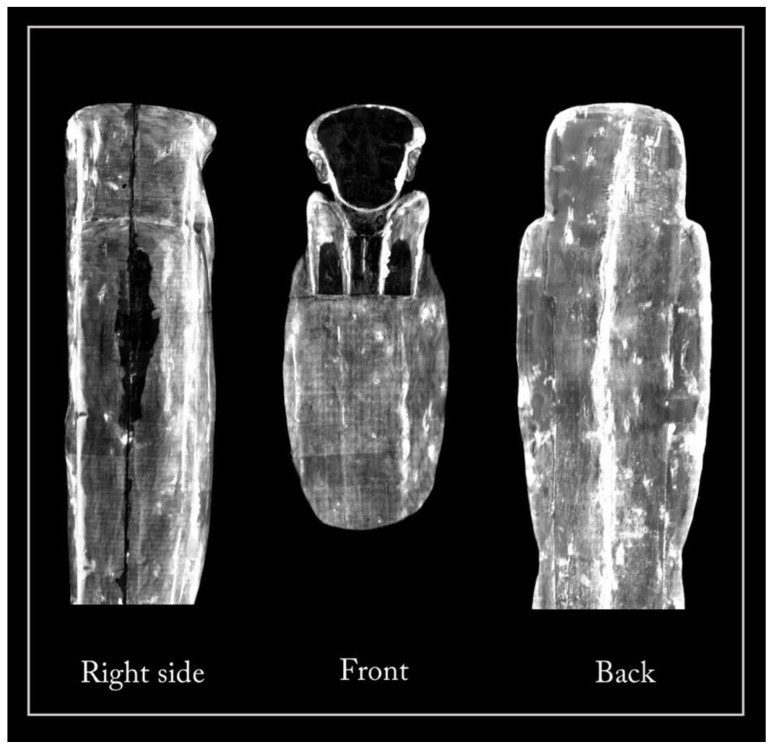
The *incamottatura* on the outer surface of the coffin.

A different tomographic display helped the legibility of this complex structure. The uniform radiopacity of the entire surface certainly derives from the thickness of this sequence of layers, in particular the stucco ground layers (Figure 15). Moreover, the penetrative power of X-rays enabled us to detect the linen canvas and understand how large bandages of textile were wrapped around the coffin structure (Figure 16). The analysis of the coffin’s exterior wall, on both sides, provided further evidence of this, thanks to the lacunae in the polychrome surface along the edges.

**Figure 17:**
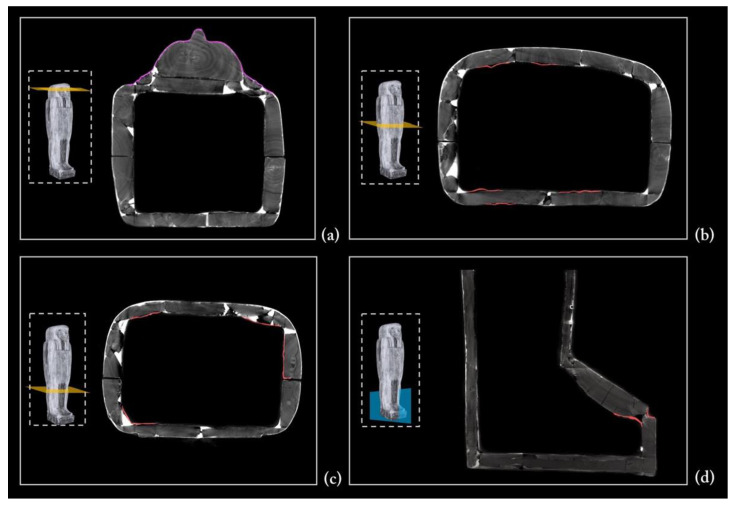
(**a**) The *incamottatura* covering face and ears (highlighted in pink); (**b**–**d**) a few examples of the *incamottatura* no longer adhering to the wooden planking surface (highlighted in red).

It is worth noting that the *incamottatura* was also used to better affix the minor wooden elements visible in the lower part of the front of the lid, as well as to keep the ears—the latter affixed with a thick layer of stucco to the planking of the lid (Figure 17a).

The canvas is also detectable in the interior wall of the coffin, lid, and box, though less homogeneous and extended. In this case, the *incamottatura* limits its function to better connect and smooth the wooden surface in the absence of a polychrome layout. It was probably created with a coarser and uneven canvas, imperfectly covering the whole interior wall of the lid and box. 

As shown in Figure 17b–d, in several areas, the *incamottatura* (highlighted in red) is no longer adherent to the wooden planking surface, owing to the shrinking and swelling of the wood in response to changes in moisture content.

- The modern restoration interventions

The CT analysis was also fundamental to our efforts to detect, specify, and quantify the interventions of a modern restoration dating back to the 1960s. 

**Figure 18:**
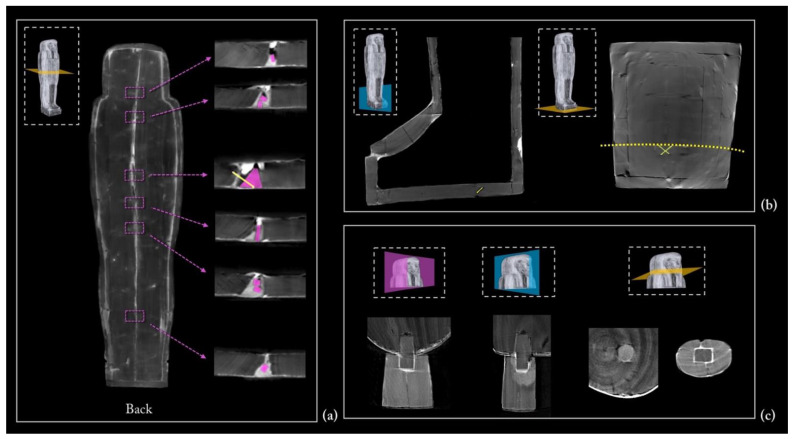
(**a**) The modern restoration interventions of the underside of the floor board, (**b**) foot board, and (**c**) beard.

A modern filler and small wooden blocks were inserted from the outside between the two planks of the floor board, which shrunk over time, in order to reinforce the coffin box (Figure 18a). The shape of these wooden elements (in purple) is completely different from the ancient dowels, and the radiopacity of the modern filler (grey areas) is different from that of the ancient stucco (white areas). A nail (in yellow) was also inserted where the distance between the two planks was greater (Figure 18a). 

Two small crossed nails were detected in the foot board near to the flat edge of the box, used to better connect a dowel and two narrow planks (Figure 18b).

Another restoration intervention that also affected the aesthetic aspect of the coffin was the joining of a modern beard to the chin by means of a square dowel, forcefully inserted into a round hole so as to crack the wood; traces of glue are also visible (Figure 18c). It is worth noting that the radiopacity of the beard wood is completely different from that of the two species detected, *Ficus sycomorus* and *Tamerix* sp (Figure 18c). The Prussian and phthalocyanine blue detected on the beard further confirm the modern intervention (the diagnostic investigations carried out before the restoration intervention attested to the nature of these pigments).

## 4. Discussion

The 3D imaging allowed us to develop hypotheses on the constructive sequence of the coffin and on the rational use of wooden planks for its manufacturing. The technologies used by the Egyptians were only apparently rudimentary because they were amply compensated by the expertise of the craftsmen who put them into practice along the centuries [78,79,80] It seems conceivable that a certain seriality was followed in the construction of this artefact, in particular in the construction of the main panels. The constructive sequence of the coffin is suggested below.

- First phase

**Figure 19:**
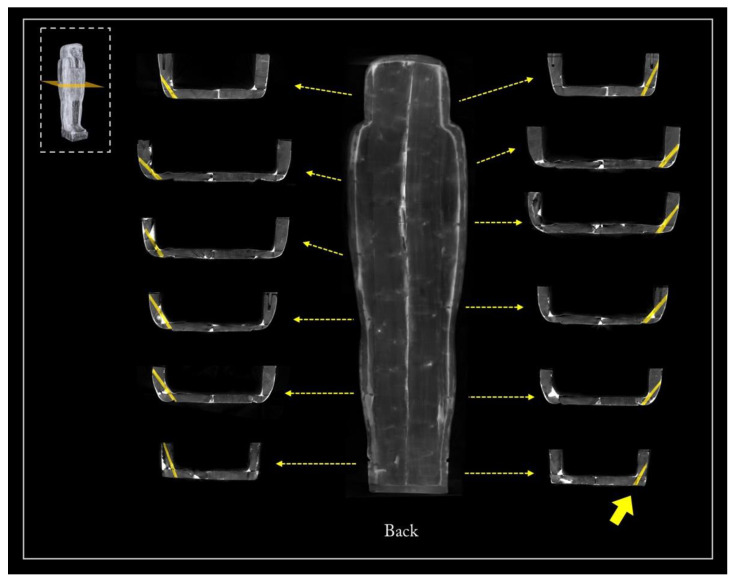
Axial sections showing the joint of the left and right side planks to the floor board by means of numerous dowels (in yellow). The arrow indicates the insertion direction of the dowels.

The coffin assembly began with the joining of the two planks of the floor board. On this planking, the single large planks of the sides were placed. The side planks and floor board were probably glued together and only later joined with long, round dowels inserted in opposing holes made in the thickness of the wood (Figure 19). 

It is worth noting again that ^14^C dating of a sample from the left side plank confirmed the well-attested practice of reusing wood to make coffins. At least, this plank dated back to the New Kingdom, 18th–19th Dynasty. Because of its size, we cannot exclude the possibility of its belonging to an outer coffin or, less probably, to another unspecified large wooden structure.

- Second phase

**Figure 20:**
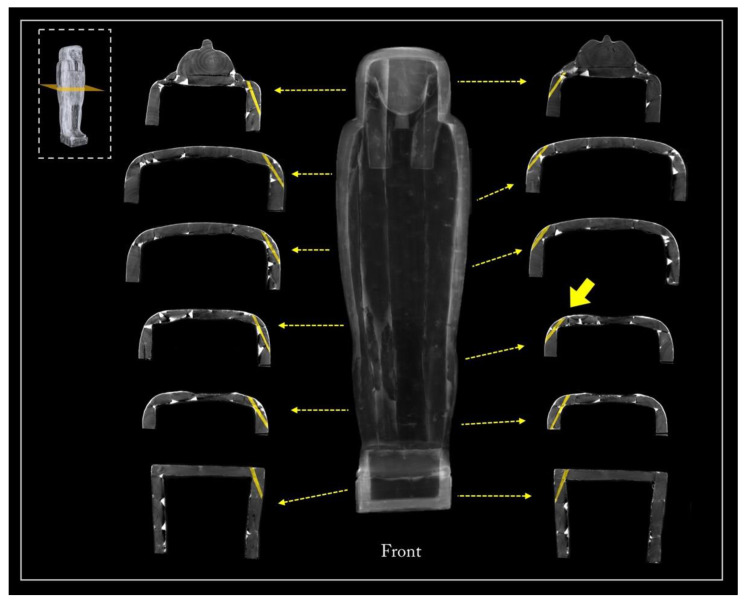
Axial sections showing the joint of the left and right side planks to the front of the lid by means of numerous dowels (in yellow). The arrow indicates the insertion direction of the dowels.

The assembly then moved on to the construction of another planking, the front of the lid, which was then affixed to the single large planks of the sides with long round dowels (Figure 20), so as to give strength to the thus obtained parallelepiped structure and make it workable with a good safety margin.

- Third phase

The parallelepiped structure obtained was then closed at the ends, adding the planking of the upper head wall, as well as the feet, pedestal, and foot board. 

First, the foot board was positioned on the floor board and joined to the side planks. Then, two smaller planks were added to each side, so as to create the profile of the feet and complete the pedestal. The next step was the application of the front plank of the pedestal. Last, the tilted plank—corresponding to the feet—that closed and sealed the coffin was applied, before sawing it into two halves. These wooden elements were probably affixed with glue and later secured with round dowels.

- Fourth phase

The next operation involved the external connection between the parts and modelling to shape the anthropoid figure and prepare the surface for the *incamottatura* and the pictorial layout. 

This process was mostly done with abrasion tools and perhaps with axes for the larger parts, then finished with sanding. In this phase of work, it is conceivable that elements too thin or broken during processing were replaced, such as the planks of the lid with many fragments or small inserts recognizable in the lower part of the legs. The abundant stucco fillings that rendered the coffin exterior uniform and connected the wooden elements to each other were laid in this phase. 

Subsequently, the face, the ears, the beard, and the wig lappets were added to complete the shaping of the coffin. The CT analysis showed that the face was slightly larger than the seat prepared on the lid planking to receive it; for this reason, it is reasonable to assume that it was applied in the final phase of the processing before applying the canvas ground layer and the *incamottatura*. 

The last part of this assembly phase involved the drafting of the *incamottatura*, which is to say, a linen canvas impregnated with protein glue (the diagnostic investigations carried out before the restoration intervention attested to the protein nature of the glue). The *incamottatura*, placed on the fresh stucco and made to adhere to the entire artefact, provided fundamental external mechanical support to the whole coffin. As shown previously in Figure 16, it consisted of fairly regular, parallel, and slightly overlapping bandages of fabric.

As for this case study, the hypothesis that the drafting of the *incamottatura* was carried out after the division of the coffin into two halves and its final closure with the body inside, as highlighted by Nicola (1989) in “*Dal museo al museo. Passato e futuro del Museo Egizio di Torino*” [81], is not supported by evidence [82,83].

- Fifth phase

**Figure 21:**
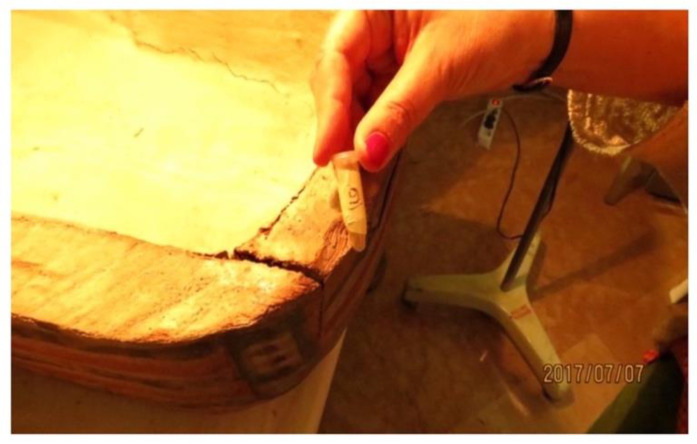
The traces of the handsaw on the surface of the flat edge of the box.

**Figure 22:**
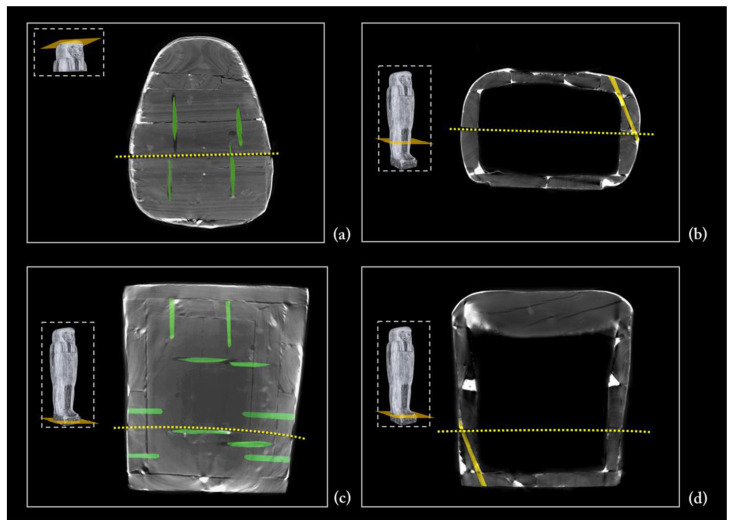
The dowels cut at the division of the coffin in the head wall (**a**), legs (**b**), foot board (**c**), and feet (**d**).

The coffin was then divided into two halves by means of a continuous cut along the established central line [81], starting from the foot board and head wall. The residual machining traces are due to sawing and abrasive tools. This operation is highlighted by a series of clues. First, it is clear that the two halves of the side planks belonged to the same original plank; this can be read from the arrangement of the growth rings in the cross sections (Figure 10). Second, traces of the handsaw are evident on the surface of both of the flat edges in which the mortises were later dug (Figure 21). Third, the canvas of the *incamottatura* was clearly continuous and was cut by the sawing of the two halves (Figure 16, right side). Finally, and most importantly, some of the dowels connecting the planks of the upper head wall (Figure 22a), the front of the lid to the right side plank (Figure 22c), as well as the floor board to the right side plank (Figure 22d) were sawn by sawing the wooden structure of the coffin into two halves. Moreover, one of the dowels connecting the foot board planks was exposed by the cut of the coffin (Figure 22b) and, consequently, fixed by means of two nails on the occasion of the modern restoration (Figure 18b).

- Sixth phase

Once the two halves of the coffin were obtained, the internal modelling was completed to define the exact inner dimensions of the coffin and grind the lateral planks to obtain an even thickness. In the same way as the exterior, the inner side was first made regular by means of a thick stucco layer, after which the *incamottatura* was applied on it to finish the preparation.

- Seventh phase

The assembly was completed with the insertion of the tenons, integrated into the lid, into the mortises, and locked by pegs to the box for final sealing. It is worth noting that the pegs go through the paint layers and the *incamottatura.*

## 5. Conclusions

The multidisciplinary investigation of Un-Montu’s coffin, with wood species analysis, radiocarbon dating, and X-ray computed tomography, unveiled the secrets of the construction technique of this ancient and complex artefact.

Furthermore, the CT disclosed the elaborated design and construction of the coffin, with the use of numerous wood planks, as well as the wisdom behind their use. Conclusively, the interconnection of all these data and the interdisciplinary approach in the data analysis enabled us to suggest the possible sequence of the coffin assembly. 

The description of the assembly phases of Un-Montu’s coffin was derived from the information obtained by diagnostic investigation in addition to thorough knowledge of the more functional sequence of actions for the construction of a wooden structure. This sequence may not correspond to a widespread and common practice. It is worth noting that this process could be typical of an area and period, of a workshop, or simply of a group of workmen, as well as a consequence of the wooden material available. Ritual implications also cannot be excluded. Further CT investigations will shed light on Egyptian coffin assembly techniques.

Moreover, it is worth noting that the application of the grouting and *incamottatura* could have been performed at different times, according to the workshop organization. The carpenters assembled, shaped, and cut the wood elements, while the decorators provided drafting of the ground layers of the polychrome layout. The question remains of whether the grouting, which had a very specific structural function for the operational sequence, could have been performed by carpenters, leaving the application of the *incamottatura* to the decorators.

The wood species identification revealed the use of two types of wood for the coffin’s construction and the radiocarbon dating proved the reuse of at least one 18th–19th Dynasty wooden plank, most likely belonging to an older coffin, which extended a well-established practice from the 19th–22nd Dynasty up to the 26th Dynasty. Additionally, the decay of this plank further confirmed the wood dating and its reuse.

Another significant fact, well attested in this case study and in many others, is the wisdom and care that went into the use of every part of the tree trunk.

This investigation focused primarily on the analysis of Un-Montu’s coffin wooden structure, the rational use of wooden planks for its manufacturing, and its construction sequence, but the sheer mass of data gained by the CT investigation still holds precious and unexpected information that could be the starting point for further studies. 

## Data Availability

Data are available from the authors with the permission of the Archaeological Museum of Bologna.

## Supplementary Materials

The wood species identification supplementary materials are available online at https://www.mdpi.com/article/10.3390/jimaging8020039/s1, Figure S1: Wood samples from the box and Figure S2: Wood samples from the lid.

## References

[1] Cavaleri T., Buscaglia P., Giudice A.L., Nervo M., Pisani M., Re A., Zucco M., Strudwick H., Dawson J. (2018). Multi and hyperspectral imaging and 3D techniques for understanding egyptian coffins. Ancient Egyptian Coffins: Past, Present, Future.

[2] Peccenini E., Albertin F., Bettuzzi M., Brancaccio R., Casali F., Morigi M.P., Petrucci F. (2014). Advanced imaging systems for diagnostic investigations applied to cultural heritage. J. Phys. Conf. Ser..

[3] Delaney J.K., Zeibel J.G., Thoury M., Littleton R., Palmer M., Morales K.M., de la Rie E.R., Hoenigswald A. (2010). Visible and infrared imaging spectroscopy of Picasso’s harlequin musician: Mapping and identification of artist materials in situ. Appl. Spectrosc..

[4] Cucci C., Casini A., Picollo M., Stefani L. (2013). Extending hyperspectral imaging from vis to NIR spectral regions: A novel scanner for the in-depth analysis of polychrome surfaces. Optics for Arts, Architecture, and Archaeology IV.

[5] Ruberto C., Mazzinghi A., Massi M., Castelli L., Czelusniak C., Palla L., Gelli N., Betuzzi M., Impallaria A., Brancaccio R. (2016). Imaging study of Raffaello’s “La Muta” by a portable XRF spectrometer. Microchem. J..

[6] Ricciardi P., Mazzinghi A., Legnaioli S., Ruberto C., Castelli L. (2019). The choir books of San Giorgio Maggiore in Venice: Results of in depth non-invasive analyses. Heritage.

[7] Darchuk L., Tsybrii Z., Worobiec A., Vázquez C., Palacios O.M., Stefaniak E.A., Gatto Rotondo G., Sizov F., Van Grieken R. (2010). Argentinean Prehistoric pigments’ study by combined SEM/EDX and molecular spectroscopy. Spectrochim. Acta Part A Mol. Biomol. Spectrosc..

[8] Prati S., Joseph E., Sciutto G., Mazzeo R. (2010). New advances in the application of FTIR microscopy and spectroscopy for the characterization of artistic materials. Acc. Chem. Res..

[9] Clark R.J.H. (2002). Pigment identification by spectroscopic means: An arts/science interface. Comptes Rendus Chim..

[10] Albertin F., Ruberto C., Cucci C., Callieri M., Potenziani M., Siotto E., Pingi P., Scopigno R., Bettuzzi M., Brancaccio R. (2021). “Ecce Homo” by Antonello da Messina, from non-invasive investigations to data fusion and dissemination. Sci. Rep..

[11] Delaney J.K., Dooley K.A., Radpour R., Kakoulli I. (2017). Macroscale multimodal imaging reveals ancient painting production technology and the vogue in Greco-Roman Egypt. Sci. Rep..

[12] Fukunaga K., Picollo M. (2012). Characterisation of Works of Art. Terahertz Spectroscopy and Imaging.

[13] Fukunaga K., Cortes E., Cosentino A., Stünkel I., Leona M., Duling I.N., Mininberg D.T. (2011). Investigating the use of terahertz pulsed time domain reflection imaging for the study of fabric layers of an Egyptian mummy. J. Eur. Opt. Soc. Rapid Publ..

[14] Abraham E., Fukunaga K. (2014). Terahertz imaging applied to the examination of artistic objects. Stud. Conserv..

[15] Monico L., Janssens K., Miliani C., Brunetti B.G., Vagnini M., Vanmeert F., Falkenberg G., Abakumov A., Lu Y., Tian H. (2012). Degradation process of lead chromate in paintings by Vincent Van Gogh studied by means of spectromicroscopic methods. 3. synthesis, characterization, and detection of different crystal forms of the chrome yellow pigment. Anal. Chem..

[16] Thurrowgood D., Paterson D., de Jonge M.D., Kirkham R., Thurrowgood S., Howard D.L. (2016). A hidden portrait by Edgar Degas. Sci. Rep..

[17] Durisi E.A., Visca L., Albertin F., Brancaccio R., Corsi J., Dughera G., Ferrarese W., Giovagnoli A., Grassi N., Grazzi F. (2013). Characterization of a neutron imaging setup at the INES facility. Nucl. Instrum. Methods Phys. Res. Sect. A Accel. Spectrometers Detect. Assoc. Equip..

[18] Andreani C., Aliotta F., Arcidiacono L., Borla M., Di Martino D., Facchetti F., Ferraris E., Festa G., Gorini G., Kockelmann W. (2017). A neutron study of sealed pottery from the grave-goods of Kha and Merit. J. Anal. At. Spectrom..

[19] Festa G., Andreani C., Baldoni M., Cipollari V., Martínez-Labarga C., Martini F., Rickards O., Rolfo M.F., Sarti L., Volante N. (2019). First analysis of ancient burned human skeletal remains probed by neutron and optical vibrational spectroscopy. Sci. Adv..

[20] Re A., Albertin F., Avataneo C., Brancaccio R., Corsi J., Cotto G., De Blasi S., Dughera G., Durisi E., Ferrarese W. (2014). X-Ray tomography of large wooden artworks: The case study of “Doppio Corpo” by Pietro Piffetti. Herit. Sci..

[21] Albertin F., Bettuzzi M., Brancaccio R., Morigi M.P., Casali F. (2019). X-ray computed tomography in situ: An opportunity for museums and restoration laboratories. Heritage.

[22] Albertin F., Bettuzzi M., Brancaccio R., Toth M.B., Baldan M., Morigi M.P., Casali F. (2020). Inside the construction techniques of the master globe-maker vincenzo coronelli. Microchem. J..

[23] Withers P.J., Bouman C., Carmignato S., Cnudde V., Grimaldi D., Hagen C.K., Maire E., Manley M., Du Plessis A., Stock S.R. (2021). X-Ray computed tomography. Nat. Rev. Methods Primers.

[24] Morigi M.P., Casali F. (2018). Radiography and Computed Tomography for Works of Art. Handbook of X-ray Imaging: Physics and Technology.

[25] Picchi D., Bertelli C., Bonsanti G. (2018). Sarcofago antropoide di unmontu. Restituzioni 2018, Tesori d’Arte Restaurati.

[26] Archivio Storico Comunale—Bologna, Scritture private 1859–1860, “Antichità Egizie in legno”, n. 35.

[27] Archivio Storico Museo Civico Archeologico—Bologna, Sub-Allegato Collezione Palagi, n. 1420.

[28] Taylor J.H., Strudwick N., Taylor J.H. (2003). Theban coffins from the twenty-second to the twenty-sixth dynasty: Dating and synthesis of development. The Theban Necropolis: Past, Present and Future.

[29] Albertin F., Baumer L.E., Bettuzzi M., Brancaccio R., Caruso E., Casali F., Cifarelli L., Festa G., Griffo M.G., Mistretta A. (2021). X-ray computed tomography to study archaeological clay and wood artefacts at lilybaeum. Eur. Phys. J. Plus.

[30] Di Turo F., Moro G., Artesani A., Albertin F., Bettuzzi M., Cristofori D., Moretto L.M., Traviglia A. (2021). Chemical Analysis and computed tomography of metallic inclusions in Roman glass to unveil ancient coloring methods. Sci. Rep..

[31] Baer F.P., Fuchs T., Wagner R., Kirsch S., Wagner R., Scholz G., Kretzer C., Schielein R., Brennhäusser G., Böhnel M. (2018). Three-dimensional computed tomography scanning of musical instruments. Wooden Musical Instruments Different Forms of Knowledge.

[32] Vigorelli L., Lo Giudice A., Cavaleri T., Buscaglia P., Nervo M., Del Vesco P., Borla M., Grassini S., Re A. Upgrade of the x-ray imaging set-up at CCR “La Venaria Reale”: The case study of an Egyptian wooden statuette. Proceedings of the 2020 IMEKO TC-4 International Conference on Metrology for Archaeology and Cultural Heritage.

[33] Maher M.A. (2020). X-ray computed tomography of a late period falcon bronze coffin. Radiat. Phys. Chem..

[34] Bettuzzi M., Casali F., Morigi M.P., Brancaccio R., Carson D., Chiari G., Maish J. (2015). Computed tomography of a medium size roman bronze statue of Cupid. Appl. Phys. A.

[35] Ciatti M., Valazzi M.R. (2015). Raffaello, “la Muta”. Indagini e Restauro.

[36] Wilson P.F., Smith M.P., Hay J., Warnett J.M., Attridge A., Williams M.A. (2018). X-Ray Computed Tomography (XCT) and Chemical Analysis (EDX and XRF) used in conjunction for cultural conservation: The case of the earliest scientifically described dinosaur megalosaurus bucklandii. Herit. Sci..

[37] Marx M., D’Auria S.H. (1986). CT Examination of eleven Egyptian mummies. RadioGraphics.

[38] Baldock C.S.W., Hughes D.K., Whittaker D.K., Taylor J., Davis R., Spencer J.R., Tonge K., Sofat A. (1994). 3-D reconstruction of an ancient Egyptian mummy using X-ray computer tomography. J. R. Soc. Med..

[39] Taylor J.H. (2004). Mummy: The Inside Story.

[40] Teeter E., Vannier M. (2009). Computed tomography scanning of Meresamun. SPIE Newsroom.

[41] Lynnerup N. (2010). Medical imaging of mummies and bog bodies–A mini-review. Gerontology.

[42] Wade A.D., Garvin G.J., Hurnanen J.H., Williams L.L., Lawson B., Nelson A.J., Tampieri D. (2012). Scenes from the past: Multidetector CT of Egyptian mummies of the Redpath Museum. RadioGraphics.

[43] Amenta A., Taylor J.H., Vandenbeusch M. (2018). New results from the CT scanning of a coffin. Ancient Egyptian Coffins. Craft Traditions and Functionality.

[44] Longo S., Mormina E., Granata F., Mallamace D., Longo M., Capuani S. (2018). Investigation of an Egyptian mummy board by using clinical multi-slice computed tomography. Stud. Conserv..

[45] Amenta A., Amenta A., Guichard H. (2017). Foreword. Proceedings of the First Vatican Coffin Conference, Città del Vaticano, 19–22 June 2013.

[46] Re A., Lo Giudice A., Nervo M., Buscaglia P., Luciani P., Borla M., Greco C. (2016). The importance of tomography studying wooden artefacts: A comparison with radiography in the case of a coffin lid from Ancient Egypt. Int. J. Conserv. Sci..

[47] Egyptian Coffins. https://egyptiancoffins.org/about.

[48] Picchi D., Bertelli C., Bonsanti G. (2013). Sarcofago antropoide di Mes-Isis o figlio di Isis. Restituzioni 2013. Tesori d’Arte Restaurati.

[49] Picchi D., Amenta A., Guichard H. (2017). The anthropoid coffin of Mesiset (?): An interesting history of collecting, typological study, and diagnostic investigation. Proceedings of the First Vatican Coffin Conference, Città del Vaticano, 19–22 June 2013.

[50] Prestipino G., Amenta A., Guichard H. (2017). The Vatican Coffin Project: Observations on the contruction techniques of third intermediate period coffins from the Musei Vaticani. Proceedings of the First Vatican Coffin Conference, Città del Vaticano, 19–22 June 2013.

[51] Bracci S., Caruso O., Galeotti M., Iannaccone R., Magrini D., Picchi D., Pinna D., Porcinai S. (2015). Multidisciplinary approach for the study of an Egyptian coffin (Late 22nd/Early 25th Dynasty): Combining imaging and spectroscopic techniques. Spectrochim. Acta Part A Mol. Biomol. Spectrosc..

[52] El-Hadidi N., Darwish S., Ragab M., Abd el-Razek S., Abd el-Rahman M., Strudwick H., Dawson J. (2018). Beyond the visible. Ancient Egyptian Coffins Past, Present, Future.

[53] Coban S.B., Lucka F., Palenstijn W.J., Van Loo D., Batenburg K.J. (2020). Explorative imaging and its implementation at the FleX-ray laboratory. J. Imaging.

[54] Natural History Museum, London, UK. https://www.nhm.ac.uk/.

[55] Lo Giudice A., Corsi J., Cotto G., Mila G., Re A., Ricci C., Sacchi R., Visca L., Zamprotta L., Pastrone N. A new digital radiography system for paintings on canvas and on wooden panels of large dimensions. Proceedings of the IEEE International Instrumentation and Measurement Technology Conference (I2MTC).

[56] Sodini N., Dreossi D., Giordano A., Kaiser J., Zanini F., Zikmund T. (2017). Comparison of different experimental approaches in the tomographic analysis of ancient violins. J. Cult. Herit..

[57] Morigi M.P., Casali F., Bettuzzi M., Brancaccio R., D’Errico V. (2010). Application of X-ray computed tomography to cultural heritage diagnostics. Appl. Phys. A.

[58] Bettuzzi M., Morigi M.P., Brancaccio R., Peccenini E., Casali F. A mobile computed tomography system for on-site cultural heritage analysis. Proceedings of the IEEE International Conference on Environment and Electrical Engineering and 2017 IEEE Industrial and Commercial Power Systems Europe (EEEIC/I&CPS Europe).

[59] Giuntini L., Castelli L., Massi M., Fedi M., Czelusniak C., Gelli N., Liccioli L., Giambi F., Ruberto C., Mazzinghi A. (2021). Detectors and cultural heritage: The INFN-CHNet experience. Appl. Sci..

[60] D’Elia M., Calcagnile L., Quarta G., Sanapo C., Laudisa M., Toma U., Rizzo A. (2004). Sample preparation and blank values at the AMS radiocarbon facility of the University of Lecce. Nucl. Instrum. Methods Phys. Res. Sect. B Beam Interact. Mater. At..

[61] Calcagnile L., Maruccio L., Scrimieri L., delle Side D., Braione E., D’Elia M., Quarta G. (2019). Development and application of facilities at the centre for applied physics, dating and diagnostics (CEDAD) at the University of Salento during the last 15 years. Nucl. Instrum. Methods Phys. Res. Sect. B Beam Interact. Mater. At..

[62] Hajdas I., Ascough P., Garnett M.H., Fallon S.J., Pearson C.L., Quarta G., Spalding K.L., Yamaguchi H., Yoneda M. (2021). Radiocarbon dating. Nat. Rev. Methods Primers.

[63] Bronk Ramsey C. (2001). Development of the radiocarbon calibration program. Radiocarbon.

[64] Brancaccio R., Bettuzzi M., Casali F., Morigi M.P., Levi G., Gallo A., Marchetti G., Schneberk D. (2011). Real-time reconstruction for 3-D CT applied to large objects of cultural heritage. IEEE Trans. Nucl. Sci..

[65] Corni E., Morganti L., Morigi M.P., Brancaccio R., Bettuzzi M., Levi G., Peccenini E., Cesini D., Ferraro A. X-ray computed tomography applied to objects of cultural heritage: Porting and testing the filtered back-projection reconstruction algorithm on low power systems-on-chip. Proceedings of the 24th Euromicro International Conference on Parallel, Distributed, and Network-Based Processing (PDP).

[66] Feldkamp L.A., Davis L.C., Kress J.W. (1984). Practical cone-beam algorithm. J. Opt. Soc. Am. A.

[67] Fedorov A., Beichel R., Kalpathy-Cramer J., Finet J., Fillion-Robin J.-C., Pujol S., Bauer C., Jennings D., Fennessy F., Sonka M. (2012). 3D Slicer as an image computing platform for the quantitative imaging network. Magn. Reson. Imaging.

[68] Schindelin J., Arganda-Carreras I., Frise E., Kaynig V., Longair M., Pietzsch T., Preibisch S., Rueden C., Saalfeld S., Schmid B. (2012). Fiji: An open-source platform for biological-image analysis. Nat. Methods.

[69] The GIMP Development Team. https://www.gimp.org.

[70] Liphschitz N. (1998). Timber identification of wooden Egyptian objects in museum collections in Israel. J. Inst. Archaeol. Tel Aviv.

[71] Asensi Amoròs M.V., Amenta A., Guichard H. (2017). The wood of the third intermediate period coffins: The evidence of analysis of the Vatican Coffin Project. Proceedings of the First Vatican Coffin Conference, Città del Vaticano, 19–22 June 2013.

[72] Abdrabou A., Abdallah M., Kamal H.M. (2017). Scientific investigation by technical photography, OM, ESEM, XRF, XRD and FTIR of an Ancient Egyptian polychrome wooden coffin. Conserv. Património.

[73] Cartwright C.R., Strudwick H., Dawson J. (2018). Identifying ancient Egyptian coffin woods from the Fitzwilliam Museum, Cambridge using scanning electron microscopy. Ancient Egyptian Coffins: Past, Present, Future.

[74] Giachi G., Guidotti M.C., Lazzeri S., Macchioni N., Sozzi L. (2021). Wood identification of some coffins from the necropolis of Thebes held in the collection of the Egyptian museum in Florence. J. Cult. Herit..

[75] Cooney K.M., Amenta A., Guichard H. (2017). Coffin reuse: Ritual materialism in the context of scarcity. Proceedings of the First Vatican Coffin Conference, Città del Vaticano, 19–22 June 2013.

[76] Cooney K.M., Strudwick H., Dawson J. (2018). Patterns of coffin reuse from dynasties 19 to 22. Ancient Egyptian Coffins: Past, Present, Future.

[77] Cooney K.M., Taylor J.H., Vandenbeusch M. (2018). Coffin reuse in dynasty 21: A case study of the coffins in the British Museum. Ancient Egyptian Coffins. Craft Traditions and Functionality.

[78] Nicola G.L., Donadoni Roveri A.M. (1989). Materiali lignei. Dal Museo al Museo. Passato e Futuro del Museo Egizio di Torino.

[79] Arbuckle C.J. (2018). A Social History of Coffins and Carpenters in Ancient Egypt. Ph.D. Thesis.

[80] MacLeod C.A., Cooney K.M. (2019). The Layered Life of JE26204: The construction and reuse of the coffins of Henuttawy. J. Egypt. Archaeol..

[81] Gale R., Gasson P., Hepper N., Killen G., Nicholson P.T., Shaw I. (2000). 15. Wood. Ancient Egyptian Materials and Technology.

[82] Buscaglia P., Cardinali M., Cavaleri T., Croveri P., Ferraris di Celle G., Piccirillo A., Zenucchini F., Strudwick H., Dawson J. (2018). Nesimenjem and the Valley of the Queen’s coffins. Ancient Egyptian Coffins: Past, Present, Future.

[83] Strudwick H., Dawson J., Marchant J., Hunkeler C., Amenta A., Guichard H. Complex layered structures on bivalve coffins. Proceedings of the Second Vatican Coffin Conference, Città del Vaticano, 6–9 June 2017.

