# Supplementary material for: X-ray Tomography Unveils the Construction Technique of Un-Montu’s Egyptian Coffin (Early 26th Dynasty)
Source: J Imaging. 2022 Feb 7;8(2):39. doi: 10.3390/jimaging8020039 (PMC8879447; doi:10.3390/jimaging8020039)
Supplement: Supplementary file 1 [file jimaging-08-00039-s001.zip › jimaging-1526858-supplementary.pdf]

Journal of
Imaging

MDPI

Supplementary Materials

- Wood species identification

As mentioned in the text, 18 samples were drawn from different portions of the coffin, both from the box and from the lid. The numbering of each sample, its position and its species can be observed in Figures S1 and S2.

All the samples drawn from the side planks (samples 1, 2, 3, 4, 5, 8, 9, 10, 13, 14, 16, 17) results of *Ficus sycomorus*. All the connecting elements, both dowels and tenons (samples 6, 7, 12, 15), were of *Tamarix* sp (probably *T. aphylla*).

The sample 11 was too small to obtain any useful information. The 18th sample was a very small wooden wedged at a mortise-tenon connection. The few available anatomical features suggested a timber from a fruity tree of the *Rosaceae* family (such as *Prunus* sp.). In this case, it could be a small element put during a modern restoration.

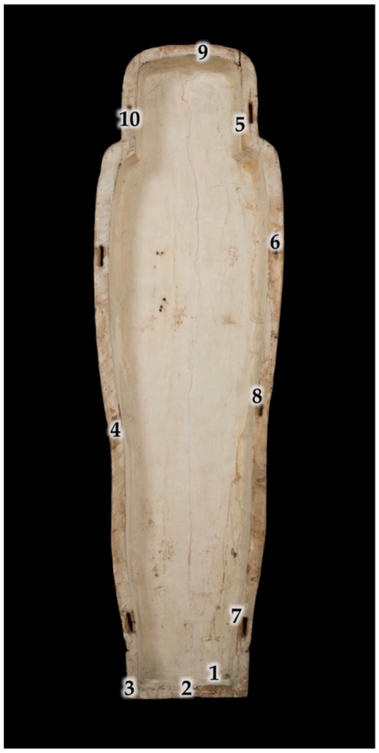

n	*Description*	*Wood*	*Species*	*Samples*
1	Coffin box, floor board, left plank	Sycamore	*Ficus Sycomorus*	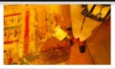
2	Coffin box, floor board, right plank	Sycamore	*Ficus Sycomorus*	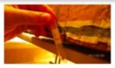
3	Coffin box, right side plank	Sycamore	*Ficus Sycomorus*	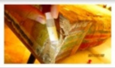
4	Coffin box, mortise, right side	Sycamore	*Ficus Sycomorus*	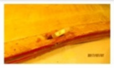
5	Coffin box, left side plank	Sycamore	*Ficus Sycomorus*	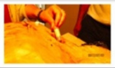
6	Coffin box, left side, dowel for mortise	Tamarisk	*Tamarix sp*	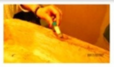
7	Coffin box, left side, tenon	Tamarisk	*Tamarix sp*	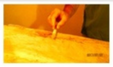
8	Coffin box, left side, mortise	Sycomore	*Ficus Sycomorus*	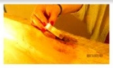
9	Coffin box, head wall	Sycomore	*Ficus Sycomorus*	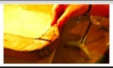
10	Coffin box, right side, mortise	Sycomore	*Ficus Sycomorus*	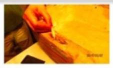

Figure S1. Wood samples from the box.

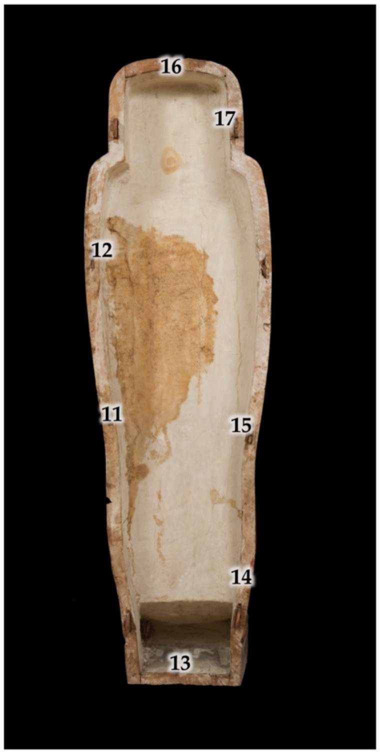

n	*Description*	*Wood*	*Species*	*Samples*
11	**Coffin lid, left side, tenon**		***Sample crumbled on boiling, probably it is rosume***	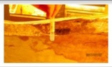
12	**Coffin lid, right side, tenon**	**Tamarisk**	***Tamarix sp***	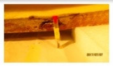
13	**Coffin lid, foot board**	**Sycomore**	***Ficus Sycomorus***	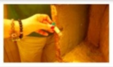
14	**Coffin lid, left side plank**	**Sycomore**	***Ficus Sycomorus***	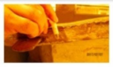
15	**Coffin lid, right side, tenon**	**Tamarisk**	***Tamarix sp***	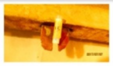
16	**Coffin lid, head wall**	**Sycomore**	***Tamarix sp***	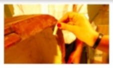
17	**Coffin lid, right side plank**	**Sycomore**	***Ficus Sycomorus***	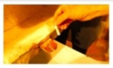

Figure S2. Wood samples from the lid.
